# Supplementary material for: Empirical methods for controlling false positives and estimating confidence in ChIP-Seq peaks
Source: BMC Bioinformatics. 2008 Dec 5;9:523. doi: 10.1186/1471-2105-9-523 (PMC2628906; doi:10.1186/1471-2105-9-523)
Supplement: Additional File 1 — A variety of html documents from the USeq web site detailing the available applications, their best usage, output file type descriptions, command line menus, etc. [file 1471-2105-9-523-S1.zip › USeqUserGuides/JavaDoc.zip!/JavaDoc/index-files/index-11.html]

L-Index


|  |  |  |
| --- | --- | --- |
| **Overview** \| Package \| Class \| Use \| **Tree** \| **Deprecated** \| **Index** \| **Help** | |  |
| **PREV LETTER**   **NEXT LETTER** | **FRAMES**    **NO FRAMES**     **All Classes** |  |


A B C D E F G H I J L M N O P Q R S T U V W X Z 

---


## **L**

**LabGroup** - Class in bioroot: **LabGroup(int, String, int)** - Constructor for class bioroot.LabGroup: **LabGroupBean** - Class in cgar.beans: Info related to a particular lab group. **LabGroupBean()** - Constructor for class cgar.beans.LabGroupBean: **LabGroupUserType** - Class in cgar: For associating a lab group with a user type. **LabGroupUserType(int, String, int)** - Constructor for class cgar.LabGroupUserType: **lauchAligner()** - Method in class gata.aligner.GATAligner: **launchCelNormalization(File[], ArrayList, File)** - Method in class trans.main.T2: **launchGATAPlotter()** - Method in class gata.main.GATAPlotter: **launchJQSub(ArrayList, int)** - Method in class trans.main.T2: **layeredLogRatios(float[][], float[][])** - Static method in class util.gen.Num: Given two float[replica][values] for treat and cont, for each replica makes all pairwise log2 ratios between treat and control. **layeredLogRatiosSeperate(float[][], float[][])** - Static method in class util.gen.Num: Given two float[replica][intensities] for treat and cont, for each oligo makes and returns all pairwise log2 ratios between treat and control replicas. **layeredRatios(float[][], float[][])** - Static method in class util.gen.Num: Given two float[replica][values] for treat and cont, for each replica makes all pairwise ratios between treat and control. **layeredRatiosSeperate(float[][], float[][])** - Static method in class util.gen.Num: Given two float[replica][intensities] for treat and cont, for each oligo makes and returns all pairwise ratios between treat and control replicas. **layeredRelativeDifferences(float[][], float[][])** - Static method in class util.gen.Num: Given two float[replica][values] for treat and cont, for each replica makes all pairwise relative differences between treat and control. **layeredRelativeDifferencesSeperate(float[][], float[][])** - Static method in class util.gen.Num: Given two float[replica][intensities] for treat and cont, for each oligo makes and returns all pairwise relative differences between treat and control replicas. **LinearRegression** - Class in util.gen: For calculating a simple linear regression. **LinearRegression(double[], double[])** - Constructor for class util.gen.LinearRegression: **ln2Log10(double)** - Static method in class util.gen.Num: Convert natural log to log 10. **ln2Minus10Log10(double)** - Static method in class util.gen.Num: Convert natural log to log 10. **loadAgilentToStandardGeneNames()** - Method in class util.bio.parsers.SynonymMatching: **loadAntibodyBean(ResultSet)** - Method in class bioroot.antibody.AntibodyBean: **loadAntibodyBean(HashMap, DBUtil, AntibodyBean, HttpServletRequest)** - Static method in class bioroot.antibody.NewAntibody: **loadAntibodyPrepBeans(HashMap, DBUtil, AntibodyBean, HttpServletRequest)** - Static method in class bioroot.antibody.NewAntibody: **loadApps()** - Method in class trans.main.T2Parameter: **loadAttribute(StringBuffer, String, String)** - Static method in class util.bio.parsers.gff.Gff3Feature: **loadAWSCredentials()** - Static method in class util.amazon.S3CodeSamples: Loads AWS Credentials from the file samples.properties (`S3CodeSamples.SAMPLES_PROPERTIES_NAME`) that must be available in the classpath, and must contain settings awsAccessKey and awsSecretKey. **loadBestWindowOligoIntensities(Interval)** - Method in class trans.main.ExportIntervalData: Loads the treatment and control intensity arrays for a particular interval. **loadBinaryBooleanArray(File)** - Method in class util.bio.seq.BinaryBooleanReaderWriter: Loads the boolean[] booleans field from a compressed binary format. **loadBinaryCoordinates(File)** - Static method in class trans.anno.Region: Reads binary chrom start stop file into Region[] **loadBinaryCoordinatesAsRegionArray(File)** - Static method in class trans.anno.RandomRegions: Reads binary chrom start stop file into Region[] **loadBPHashMap(int, File)** - Method in class trans.tpmap.TPMapDuplicateFilter: **LoadChipSetIntervalOligoInfo** - Class in trans.main: Loads arrays of Interval[] with oligo and sequence information needed for other applications. **LoadChipSetIntervalOligoInfo(String[])** - Constructor for class trans.main.LoadChipSetIntervalOligoInfo: **loadChromosomeBarFile()** - Method in class trans.graphics.AggregatePlots: **loadChromosomeBarFile()** - Method in class trans.roc.ScoreParsedBars: **loadChromosomeGrs(String)** - Method in class trans.roc.ExonScoreExtractor: **loadContactInfo(DBUtil)** - Method in class bioroot.ContactBean: **loadCredentials()** - Method in class util.amazon.S3Manager: Attempts to create a AWSCredentials object from an encripted file. **loadDoubleMatrix(File)** - Static method in class util.gen.Num: Loads matrix of double[row number][double columns], white space delimited. **loadDoubles(File, boolean)** - Static method in class util.gen.Num: Loads columns of double into a HashMap, the first row is parsed as Strings and used as the key. **loadDoubles(File)** - Static method in class util.gen.Num: Loads a column of double from a file into an array. **LoadedOligo** - Class in trans.bkgrnd: BPMap line contain gc and tm calculations. **LoadedOligo(String, int, String, double, double, double, double)** - Constructor for class trans.bkgrnd.LoadedOligo: **LoadedOligo(String)** - Constructor for class trans.bkgrnd.LoadedOligo: **loadField(StringBuffer, String)** - Static method in class util.bio.parsers.gff.Gff3Feature: **loadFile(File)** - Static method in class bioroot.parsers.ExtractWords: Loads a file's lines into a String[], will save blank lines. **loadFile(File)** - Static method in class bioroot.parsers.RenameWords: Loads a file's lines into a String[], will save blank lines. **loadFile(File)** - Static method in class util.apps.GeneNameCounter: Loads a file's lines into a String[], will save blank lines. **loadFile(File)** - Static method in class util.gen.IO: Loads a file's lines into a String[], will save blank lines. **loadFileIntoHashMap(File)** - Static method in class util.gen.IO: Loads a file's lines into a hash first column is the key, second the value. **loadFileIntoHashSet(File)** - Static method in class bioroot.parsers.ExtractWords: Loads a file's lines into a hash set, keys only. **loadFileIntoHashSet(File)** - Static method in class util.gen.IO: Loads a file's lines into a hash set, keys only. **loadFileIntoHashSplitLines(File)** - Static method in class util.gen.IO: Loads a file's lines into a hash. **loadFileIntoKeyValueStrings(File)** - Static method in class bioroot.parsers.RenameWords: **loadFileIntoLinkedHashSet(File)** - Static method in class util.gen.IO: Loads a file's lines into a linked hash set, keys only. **loadFileIntoStringArray(File)** - Static method in class util.gen.IO: Loads a file's lines into a String[] trimming blank lines and beginning and ending whitespace. **loadFloatArray(int, int, float)** - Static method in class util.gen.Num: Makes a float[x][y] and fills it with the value. **loadFloatArrays(boolean)** - Method in class trans.qc.CelFileStats: Loads the serialized virtualCel (float[][]) from disk and then collapses it to generate the intensities (float[]). **loadFloats(File)** - Static method in class util.gen.Num: Loads a column of float from a file into an array. **loadGeneral()** - Method in class trans.main.T2Parameter: **loadGrArray(Gr[])** - Method in class trans.misc.GrGraph: **loadGrFile(File)** - Static method in class trans.misc.CorrelateGrFiles: Reads in a gr file returning it's float values. **loadGrFile(File, String)** - Static method in class trans.misc.Util: Reads in an sgr file. **loadHash(File)** - Static method in class util.gen.IO: Loads a tab delimited file into a LinkedHashMap where each line is broken into cells and loaded into a LinkedHashMap where the cells are the keys and the value is the line. **loadHash(String[])** - Static method in class util.gen.Job: Converts a String[] of key = values into a HashMap. **loadHashSet(Object[])** - Static method in class util.gen.Misc: Loads a HashSet with and Object array. **loadHits(String)** - Method in class edu.utah.seq.parsers.ElandMultiParser: **loadIntensities(BackGroundOligo[], File[])** - Static method in class trans.bkgrnd.MapControlIntensities: **loadIntensities(File, String)** - Static method in class trans.main.LoadChipSetIntervalOligoInfo: Loads the oligo intensities. **loadIntensityArrays(File[])** - Method in class trans.cel.QuantileNormalization: Fetch intensity arrays from .celaMap files. **loadIntervalArrays()** - Method in class trans.main.T2: Creates set number of intervals. **LoadIntervalOligoInfo** - Class in trans.main: Loads arrays of Interval[] with oligo and sequence information needed for other applications. **LoadIntervalOligoInfo(String[])** - Constructor for class trans.main.LoadIntervalOligoInfo: **loadIntervals(File)** - Method in class trans.main.ExportIntervalData: **loadInts(File)** - Static method in class util.gen.Num: Loads a column of ints from a file into an array. **loadKeyValueFile(File)** - Static method in class util.gen.IO: Loads a key = value containing text file into a LinkedHashMap. **loadKeyValueSpaceTabDelimited(File, boolean)** - Static method in class util.gen.IO: Loads a key value containing text file into a LinkedHashMap. **loadMapsAndRawCelFiles()** - Method in class trans.main.T2Parameter: **loadMatrixValues(float[][], int[][])** - Static method in class util.gen.Num: Given a square table and a list of coordinates, will return the associated values in a new zeroed square table. **loadMissingChromosomes()** - Method in class edu.utah.seq.analysis.QCSeqs: **loadNewURL(String)** - Method in class gata.main.TextWindow: **loadNumberOligos()** - Method in class trans.main.ChromSet: **loadOligoBean(ResultSet)** - Method in class bioroot.oligo.OligoBean: **loadOligoPositions(String)** - Method in class trans.main.LoadChipSetIntervalOligoInfo: Loads the oligo positions. **loadOrganizationInfo(DBUtil, int)** - Method in class bioroot.Organization: **loadParamFile()** - Method in class util.apps.JQSub: **loadPlasmidBean(ResultSet)** - Method in class bioroot.plasmid.PlasmidBean: **loadPositionScores()** - Method in class edu.utah.seq.data.PointData: Gets the BarParser to (re)load the position and scores. **loadPositionValues(int[])** - Method in class edu.utah.seq.parsers.Tag2Point: **loadReagentBean(ResultSet)** - Method in class bioroot.ReagentBean: **loadRegion(Positive)** - Method in class trans.roc.ScoreParsedBars: Loads a Positive with Gr objects. **loadRegions(Sgr, Positive[])** - Static method in class trans.graphics.AggregatePlots: Checks to see if an sgr object is in the positives, if so, adds the sgr object to the positive. **loadRegions(Sgr, Positive[])** - Static method in class trans.roc.ParseSgrsForParticularRegions: Checks to see if an sgr object is in the positives, if so, adds the sgr object to the positive. **loadRemainingFields()** - Method in class cgar.beans.UserBean: Loads **loadResults(HashMap)** - Method in class util.bio.wrappers.Primer3Pick: Makes a tab delimited results line: PRIMER\_LEFT\_SEQUENCE PRIMER\_RIGHT\_SEQUENCE PRIMER\_LEFT\_TM PRIMER\_RIGHT\_TM PRIMER\_PRODUCT\_SIZE The user entered line If no results then leaves no picks message. **loadRocWindows(File[])** - Static method in class trans.roc.ParsePatternedWindows: Loads all the RocWindows into one from serialized ArrayLists of RocWindows. **loadScores(File[], String)** - Method in class util.apps.GeneCentricCNVParser: **loadSequences()** - Method in class util.bio.parsers.FetchGenomicSequences: **loadSgrFile(File)** - Static method in class trans.misc.GrGraph: Loads an sgr file returning chromosome specific GrGraphs. **loadSgrFile(File)** - Static method in class trans.misc.Util: Reads in an sgr file. **loadSgrFile(File)** - Static method in class trans.roc.Sgr: Loads an xxx.sgr(.zip) file into an ArrayList of Sgr **loadSimpleBarFile(boolean)** - Method in class edu.utah.seq.parsers.BarParser: This is a complete hack of Gregg Helt's IGB code to parse bar files. **loadStrainBean(ResultSet)** - Method in class bioroot.strain.StrainBean: **loadSuperUserEmailAddresses(DBUtil, LabGroup[])** - Static method in class bioroot.CronArchive: Loads an array of LabGroup[] with superuser email addresses. **loadTableOfInts(File)** - Static method in class util.gen.IO: Loads a tab delimited file containing ints into an int[columns][rows], all lines included, assumes no missing values. **loadTVSHash()** - Method in class trans.cel.ConvertTVSToCelps: **loadTxtRocFile(File)** - Static method in class trans.roc.ParsePatternedWindows: reads a txt roc window (chrom, start, stop, middle(not used), score). **loadUseDilution(ResultSet)** - Method in class bioroot.antibody.UseDilution: **loadUseDilutions(HashMap, AntibodyPrep, int, HttpServletRequest)** - Static method in class bioroot.antibody.NewAntibody: **loadWorkingFile()** - Method in class edu.utah.seq.parsers.ElandParser: Splits a tag file by chromosome and strand to seperate files. **loadWriteBinaryCoordinates(File)** - Static method in class trans.anno.RandomRegions: Looks for a binary version of the file xxx.corr, if found loads binary, otherwise it parses the txt file and then writes the binary for future use. **loadWriteBinaryRegions(File)** - Static method in class trans.anno.Region: Looks for a binary version of the file xxx.corr, if found loads binary, otherwise it parses the txt file and then writes the binary for future use. **LocalAlignment** - Class in gata.aligner: **LocalAlignment(String, int, int, String, int, int, int, AlignParams, int)** - Constructor for class gata.aligner.LocalAlignment: **log** - Static variable in class bioroot.Organization: **log** - Static variable in class bioroot.ReagentBean: **log10** - Static variable in class util.gen.Num: **log10(double)** - Static method in class util.gen.Num: Returns the LOG base 10 of the number. **log2** - Static variable in class util.gen.Num: **log2(float[][])** - Static method in class util.gen.Num: Log2s every number in the array replacing the original values. **log2(float[])** - Static method in class util.gen.Num: Log2s every number in the array replacing the original values. **log2(double[])** - Static method in class util.gen.Num: Log2s every number in the array replacing the original values. **log2(double)** - Static method in class util.gen.Num: Returns the LOG base 2 of the number. **log2(float)** - Static method in class util.gen.Num: Returns the LOG base 2 of the number. **log2Return(float[][])** - Static method in class util.gen.Num: Log2s every number in the array returning the values, keep original intact. **log2Return(float[])** - Static method in class util.gen.Num: Log2s every number in the array keeping original intact. **log2ReturnDouble(float[])** - Static method in class util.gen.Num: Log2s every number in the array keeping original intact. **logEm(int[])** - Static method in class util.apps.ScatterDrawPanel: **Login** - Class in bioroot: **Login()** - Constructor for class bioroot.Login: **Login** - Class in cgar: **Login()** - Constructor for class cgar.Login: **LoginBean** - Class in bioroot: **LoginBean()** - Constructor for class bioroot.LoginBean: **LoginBean(String, String)** - Constructor for class bioroot.LoginBean: **LoginBean** - Class in cgar.beans: **LoginBean()** - Constructor for class cgar.beans.LoginBean: **LoginBean(String)** - Constructor for class cgar.beans.LoginBean: **Logout** - Class in bioroot: Logout page, kills session, frees objects **Logout()** - Constructor for class bioroot.Logout: **logRatios(double[], double[])** - Static method in class util.gen.Num: Calculates log base 2 ratios. **logRatios(float[], float[])** - Static method in class util.gen.Num: Calculates log base 2 ratios. **logRatios(float[][], float[][])** - Static method in class util.gen.Num: Returns the log2 ratio of each pair, averaging replicas. **lotsOfNs(String)** - Method in class edu.utah.seq.data.Simulator: Check N content. **lotsOfRepeats(int, int)** - Method in class edu.utah.seq.data.Simulator: Checks repeat content **LowerCase2Xs** - Class in util.bio.seq: Converts a directory of files that contain lowercase masked sequences to Xs. **LowerCase2Xs()** - Constructor for class util.bio.seq.LowerCase2Xs

---


|  |  |  |
| --- | --- | --- |
| **Overview** \| Package \| Class \| Use \| **Tree** \| **Deprecated** \| **Index** \| **Help** | |  |
| **PREV LETTER**   **NEXT LETTER** | **FRAMES**    **NO FRAMES**     **All Classes** |  |


A B C D E F G H I J L M N O P Q R S T U V W X Z 

---
